# The iPlant Collaborative: Cyberinfrastructure for Enabling Data to Discovery for the Life Sciences

**DOI:** 10.1371/journal.pbio.1002342

**Published:** 2016-01-11

**Authors:** Nirav Merchant, Eric Lyons, Stephen Goff, Matthew Vaughn, Doreen Ware, David Micklos, Parker Antin

**Affiliations:** 1 The University of Arizona, Tucson, Arizona, United States of America; 2 Texas Advanced Computing Center, The University of Texas at Austin, Austin, Texas, United States of America; 3 Cold Spring Harbor Laboratory, Cold Spring Harbor, New York, United States of America

## Abstract

The iPlant Collaborative provides life science research communities access to comprehensive, scalable, and cohesive computational infrastructure for data management; identity management; collaboration tools; and cloud, high-performance, high-throughput computing. iPlant provides training, learning material, and best practice resources to help all researchers make the best use of their data, expand their computational skill set, and effectively manage their data and computation when working as distributed teams. iPlant’s platform permits researchers to easily deposit and share their data and deploy new computational tools and analysis workflows, allowing the broader community to easily use and reuse those data and computational analyses.

## Abstract 2

This Community Page highlights the iPlant Collaborative, which provides scalable and extensible data management and computational resources for life science research, including learning materials and support to readily integrate these resources with existing workflows.

Over the last decade, the discipline of life sciences has benefited tremendously from new, massively parallel, and highly quantitative technologies. These technologies have facilitated rapid data acquisition at an increasingly higher resolution and throughput across all forms of modalities, from super-resolution microscopy to DNA sequencing technologies. Transformational advances in information technology have complemented this phenomenal growth in data acquisition, including cloud and high performance computing, large-scale data management systems, and high-bandwidth networks. However, managing the life cycle of these datasets from acquisition and analysis to publication and archiving often necessitates interdisciplinary collaborations with geographically distributed teams of experts. A common requirement for these interdisciplinary teams is access to integrated computational platforms that are flexible, scalable, and agile. These platforms must provide access to appropriate hardware and software that support diverse data types, computational scalability needs, and the usage patterns of diverse research communities. This includes access to shared data storage that can reliably transfer large sets of data, ability to annotate and search these data with descriptive metadata, connections to appropriate computational hardware (e.g., high-memory computers, virtual machines) for analysis, and identity management systems to securely share data with collaborators. The iPlant Collaborative, a National Science Foundation (NSF) funded cyberinfrastructure (CI) project launched in 2008, is meeting the needs associated with managing data-driven research, collaborations, and discoveries. While originally targeted toward the plant science research community, iPlant has the expanded mandate to provide CI support across the life sciences.

To meet the needs of rapidly expanding and diverse user communities, iPlant provides access to a comprehensive and cohesive suite of computational resources supporting data management, cloud computing, high-performance computing, high-throughput computing, identity management, and collaboration tools. In addition, iPlant provides training resources, knowledge bases, and examples of best practices to help all researchers make the best use of their data, expand their computational skill set, work with a distributed team, and identify new collaborators. iPlant’s platform is built from open source components, integrating multiple well-established and proven foundational platforms and technologies that have broad adoption in both academia and industry. In order to link these resources together, iPlant developed an extensive set of middleware, which are available for programmers and computational researchers to use to access the underlying technologies directly. iPlant’s CI takes inspiration from exemplar science gateways, such as Galaxy [1] and HubZero [2], that provide software infrastructure for establishing domain specific CI components. Similar to those platforms, iPlant lets programmers and computational researchers easily deploy new analytical tools for others to use. However, iPlant’s expands on these types of software infrastructure projects by also providing access to underlying data management and computing hardware, including providing the ability to extend its capacity by creating complete computational appliances (complete software systems including the operating system and all specialized software) and federating with computational resources from other infrastructure providers. These CI resources are accessible using multiple methods, which include Web-accessible applications for ease of use, command-line–based access, and well-described Application Programming Interfaces (APIs) for ease of automation and performing scalable data analysis. Together, iPlant’s CI permits researchers to deposit and share new data, programmers to easily deploy new tools and analytical workflows, and researchers of all skill levels to easily use and reuse those data and tools.

iPlant’s CI architecture and implementation is agnostic with regards to scientific domain and supports many different life science disciplines and their associated data types and analyses. iPlant’s CI allows research communities to readily utilize a wide array of tools and services, and permits them to extend and customize the CI to accommodate their community’s specific needs by creating shared data resources, integrating new tools, and coordinating documentation. This innate flexibility allows diverse scientific communities, global consortiums, and individual investigators to effectively adopt and utilize relevant components from iPlant’s CI for their project-specific needs. iPlant provides an avenue for researchers to share their research data, software tools, analysis pipelines, and best practices with their collaborators and/or a large community of users without burdening the individuals with managing the underlying computational infrastructure. iPlant’s CI introductory orientation and learning materials are available as asynchronous online tutorials, monthly Webcasts, YouTube videos, Twitter feeds, and online support forums, with onsite workshops and boot camps held on a regular basis. For projects requiring sustained engagement, communities can request Extended Collaborative Support (ECS) that pairs iPlant staff members with community partners for addressing specific computational aspects for their project. Table 1 highlights a subset of exemplar community initiated projects and activities that utilize various components of iPlant CI.

**Table 1:** Subset of exemplar community-initiated projects and activities that utilize various components of iPlant CI.

Application Domain	User Community	iPlant CI components utilized
Data sharing and analysis platform to support regional and international collaborations	Integrated Breeding Platform (IBP): https://www.integratedbreeding.net	Auth, Data Store, Discovery Environment, Atmosphere
	Genomes to Fields Initiative: http://www.genomes2fields.org/	
Large-scale image data management	120+ Herbariums, part of NEVP (New England Vasculature Plant) and SERNEC (Southeast Region Network of expertise and collections) consortium: http://nevp.org/participants, http://sernec.appstate.edu/	Auth, Data Store, Bisque
High-throughput Image based phenotyping	15 laboratories in the United States and Mexico collaboratively working with robotic data acquisition systems and cloud-based analysis using customized virtual machines (appliances)	Auth, Data Store, Atmosphere
High-throughput Next Generation Sequencing analysis pipelines	Workflows with 100+ TBs of data, Millions of CPU hours	Auth, Data Store, Discovery Environment, Atmosphere, Pegasus [3], Science API
	iAnimal variant calling workflows	
	Soybean Knowledgebase (SoyKB): http://soykb.org/	
Community portals for data dissemination	Integrated large-scale data sets and analysis	Auth, Data Store, Discovery Environment
	iMicrobe (includes legacy CAMERA project data): http://imicrobe.us	
	One thousand plants transcriptome: http://onekp.com	
Learning Material, training opportunities	Undergraduate course work: http://www.iplantcollaborative.org/blog/news/class-cloud	DNA subway, Atmosphere, Discovery Environment, Science API
	Graduate Bioinformatics courses: http://brendelgroup.org/teaching/2014/CGSL14Sschedule.php	
	Community organized workshops and data clinics: http://www.idsnews.com/article/2015/08/iu-biology-hosts-clinic	
	Software and Data Carpentry with iPlant: http://dib-training.readthedocs.org/en/pub/2015-09-iplant.html	
CISE (Computer Information Science and Engineering) engagement	Jet Stream: Self-provisioned cloud environment: http://jetstream-cloud.org/	Atmosphere, Data Store, Science API
	Syndicate: Content Delivery Network (CDN) for science data: http://www.nsf.gov/awardsearch/showAward?AWD_ID=1541318	
	Agave: API driven platform for reproducible science: http://www.nsf.gov/awardsearch/showAward?AWD_ID=1450459	

## iPlant CI Overview

**Fig 1:**
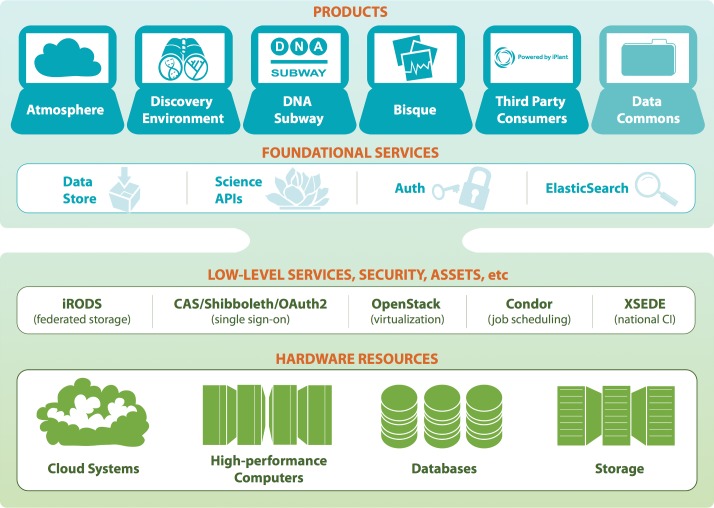
iPlant CI architecture and technology components. Image credit: Monica Lent.

iPlant’s CI is made up of several functional layers that are built on top of one another (Fig 1). At the foundational level are the primary hardware resources (storage, compute), which are transformed into low-level services using software components developed by projects such as iRODS (data management) [4], Condor (job execution) [5], CAS [6] and Shibboleth [7] (user identity management and authentication), and Openstack [8] (cloud computing infrastructure). These two foundational levels are accessible to advanced users that are well versed in direct use of these computational resources and wish to automate, augment, and scale their analyses. Utilizing these low-level services, iPlant has developed novel foundational services that abstract away the underlying infrastructure complexities, thus allowing rapid development of new community-facing products as well as permitting existing bioinformatics platforms the ability to integrate with existing iPlant products. The architecture of iPlant’s CI is designed so that researchers can access every level, with the goal that the lower levels are more flexible, while the upper levels are easier to use (e.g., programming and command-line interfaces versus graphical interfaces). In addition to providing training on using iPlant’s CI, iPlant’s training resources also assist researchers in transitioning from one layer of iPlant’s CI (graphical interfaces) to another (command-line and programming interfaces).

### Foundational Services

**Fig 2:**
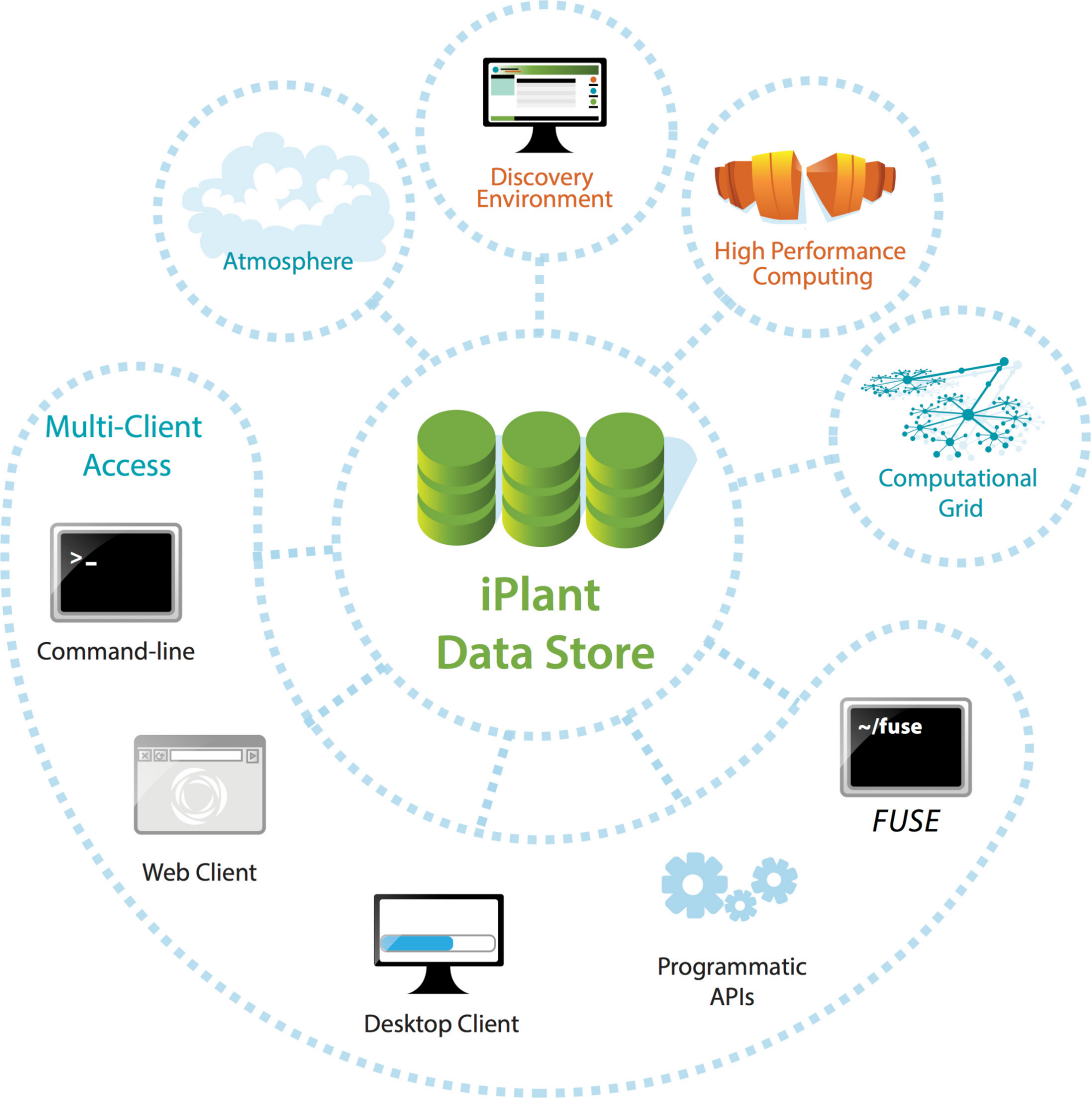
iPlant Data Store: Fabric that connects multiple CI components. Image Credit: Monica Lent.

Extending the foundational infrastructure level, iPlant has built foundational services, such as the **iPlant Data Store** that provides scalable data management capabilities across all of iPlant’s CI. The iPlant Data Store can be used directly by individual researchers as well as by third-party, independent platforms (Fig 2). By enabling access through various methods, novice and advanced users have the means to manage datasets ranging from a few megabytes to multiple terabytes in size. All researchers can efficiently upload and download data using parallel data transfer methods from graphical interfaces or the command line, and securely share datasets with collaborators. Data types such as BAM and GFF can be directly integrated with popular genome browsers (UCSC, ENSEMBL, JBrowse, etc.) Computationally savvy users can use advanced methods to develop programs to automate data management tasks, such as after a large image file is uploaded, extract its metadata and alert the image processing services to process the file for viewing and analysis in the Bisque platform (described below). The iPlant Data Store also provides robust support for metadata, ensuring that data can be associated with appropriate provenance, ontology, or other information that describes its history.

A core middleware developed by iPlant is its identity management system (**iPlant Auth**). This system is essential to ensure data privacy and sharing for all researchers. In addition, having a single sign-on (SSO) identification system allows users to navigate seamlessly across all of iPlant’s resources as well as registered third-party and externally hosted projects. Once users are authenticated to any of these SSO-enabled services, they can access other resources without having to re-authenticate. For advanced computational users, iPlant’s **Science API** provides RESTful Web APIs to iPlant’s resources. These APIs are used to automate use of iPlant’s data and computational resources, and are frequently used to integrate third party bioinformatics platforms and develop new applications. The metadata and content associated with iPlant’s CI is indexed using **Elasticsearch** [9], which provides secure and scalable search and discoverability of data at petabyte scales and indexes the tens of millions of metadata attributes currently stored in the data store.

### Community-Facing Products

Community-facing products are made available as easy-to-use, Web-based applications and form iPlant’s vibrant and diverse ecosystem of interoperable applications. These are integrated using the underlying foundational services such as iPlant Auth and iPlant Data Store.

**Atmosphere** provides access to iPlant’s cloud computing infrastructure, and addresses complexities associated with managing the lifecycle of cloud images, virtual machines (VM), and running VM instances. Through Atmosphere’s Web interface, researchers can easily launch, provision, manage, build, and share customized virtual machines that include complete software dependencies for running complex applications. For example, a researcher can deploy a new genome assembly visualization tool that includes additional tools for cleaning and assembling sequencing reads by different methods. When this virtual machine is made available, another researcher can launch it, connect to it, move their data from the iPlant Data Store, analyze those data, and send the results back to the Data Store. In addition, they can install new software to expand the analysis options and create a new virtual machine image for others to launch and use. Atmosphere supports Web, command-line, and graphical user interface-based Linux applications, and allows researchers to share a desktop on a running instance for real-time collaborative analysis. Central to iPlant’s efforts to support reproducibility, Atmosphere provides the ability to create snapshots of a running instance, encapsulating all software on it, and sharing the new version with any researcher to reuse and reproduce analyses. This method is widely utilized by iPlant users to distribute complex applications, e.g., high-throughput image phenotyping as ready-to-use virtual machines. This functionality provides a reliable and consistent software environment for research teams, students, and educators, making Atmosphere an attractive platform for special topic workshops, bioinformatics courses, and analyses that require adherence to Standard Operating Procedures (SOPs), while minimizing the complexities typically associated with installing complex software, user management, network security, and image catalogs when using traditional cloud services.

**Fig 3:**
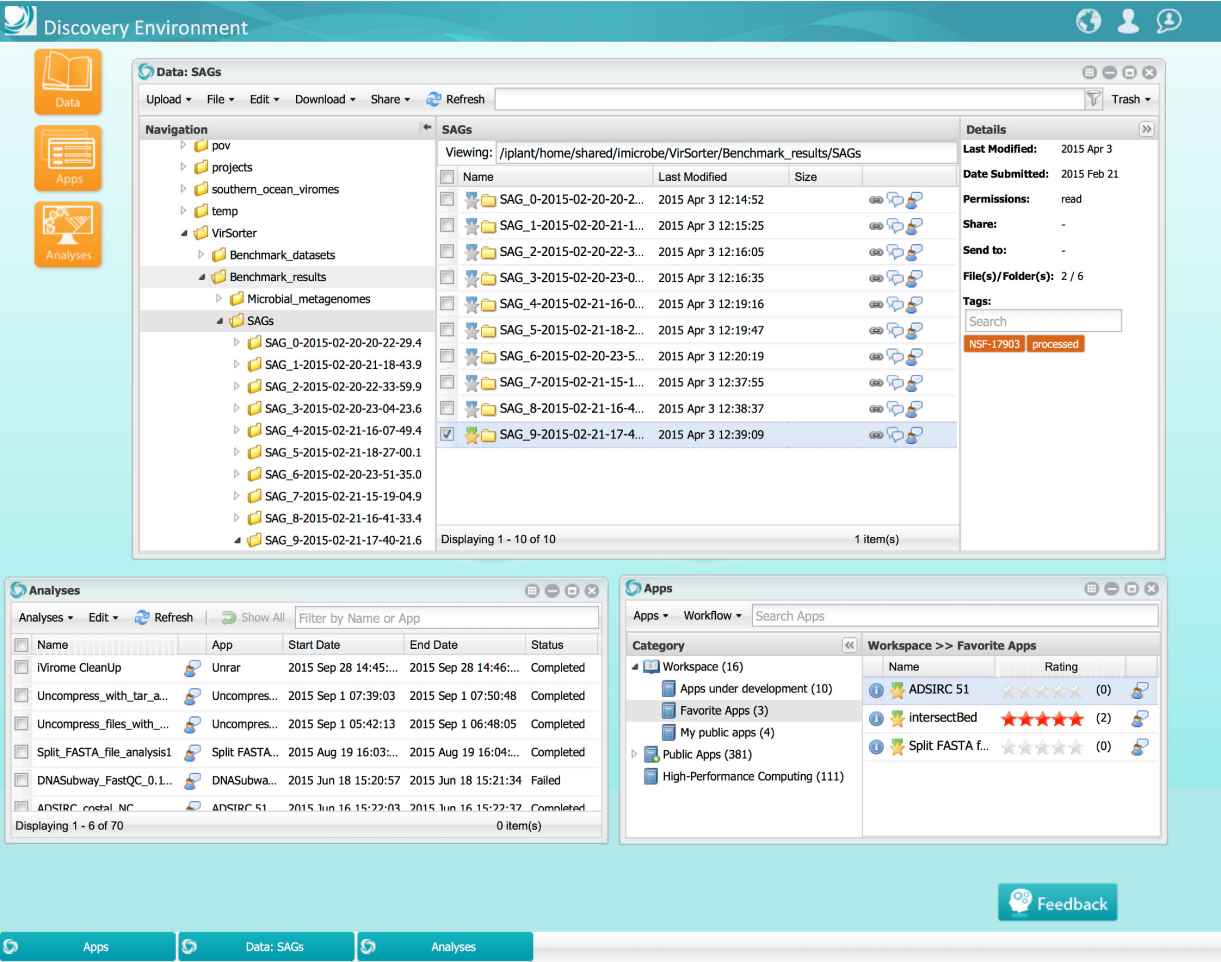
iPlant Discovery Environment, showing the Data, App, and Analyses windows.

The **Discovery Environment** (DE) provides a Web-based workbench for analysis and data management tasks. The DE interface (Fig 3) provides a catalog of popular analysis tools (apps), with the ability for researchers to rapidly integrate new Linux-based command-line programs and create a web interface to it. All applications available in the DE can access data available in the iPlant data store and can connect with external providers for computational scalability (e.g., XSEDE) using the iPlant Science API. Data management tasks in the DE are accessible through the Data window; these include sharing and access control, metadata templates and advanced search capabilities. The DE natively supports the use of Docker [10] based virtual images and software environments for integrating new apps, allowing researchers to readily incorporate their custom applications, scripts, and utilities into iPlant. Utilizing Docker capabilities researchers can develop, prototype, and test applications in their choice of operating systems and familiar development environments, and share them using iPlant CI without additional programming effort. In addition, the DE also connects with external data and service providers such as National Aeronautics and Space Administration (NASA)’s MERRA A/S systems [11] (30-plus years of climate observational data and numerical models), providing seamless integration with large data repositories, and iPlant’s tool integration and analysis interfaces.

**DNA Subway** uses an intuitive metaphor of a subway map to provide access to configured, high-level genome analysis workflows targeted at students and educators. DNA Subway organizes research-grade bioinformatics analysis tools into logical workflows that educators can readily employ as part of their learning material. DNA Subway leverages iPlant’s Science API to access scalable computing and data management resources, permitting students to easily transition to more complex, powerful, and flexible parts of iPlant’s CI.

**Bisque** is an integrated platform for managing, analyzing, and visualizing high-throughput image data. It supports interactive viewing and annotation of large (100,000 × 100,000 pixel) 2-D, 3-D, 4-D, and 5-D image stacks using a Web browser. It also supports importing and exporting of videos. Bisque leverages iPlant Auth and Data Store, providing the means for researchers to easily manage large sets of image data and share them with collaborators.

The **Powered by iPlant** program facilitates **third-party applications and developers** to rapidly build on iPlant’s foundational services, incorporating scalability into existing applications by offloading resource-intensive tasks to iPlant. It also provides seamless access to external resources for iPlant users without requiring them to create separate accounts and registering at each service provider. This layer of user account federation also ensures that multiple third-party applications can securely read and write data to the iPlant Data Store on behalf of users. This initiative supports many community-led efforts such as iAnimal, iMicrobe, iVirus, and CoGe, which together with iPlant are rapidly creating a vibrant ecosystem of informatics resources. While each platform retains its unique identify, user community, and interfaces, by leveraging iPlant’s CI, they gain computational scalability and interoperability.

The iPlant **Data Commons** provides the tools and best practices to help researchers rapidly publish data with requisite metadata standards, encouraging reuse and discoverability through digital object identifiers (DOIs). The Data Commons includes high-throughput data deposition to canonical repositories such as NCBI’s Short Read Archive (SRA) directly from iPlant Data Store with full metadata support and the ability to expose these submitted datasets to institutional repositories.

In addition, iPlant strives to provide access to its CI for researchers and students with varying levels of computational skills and expertise, providing extensive learning material and avenues for obtaining hands-on scientific and technical computing skills for effectively utilizing CI resources provided by iPlant and other providers. iPlant actively partners with organizations such as Software [12] and Data Carpentry [13] to offer command-line and scripting boot camps and data management best practices workshops, as these are essential and foundational skills required for working with large-scale data and analyses tasks.

We invite the life-science research community to explore iPlant’s tools and services as both users and contributors to the platform. We encourage community feedback and input for refining, improving, and extending iPlant’s capabilities that can scale to today’s research challenges and future discovery opportunities. iPlant actively collaborates with the Computer Information Science and Engineering (CISE) research communities for incorporating the emergent CI needs identified by iPlant users into national CI systems currently being designed and deployed. iPlant’s CI will continue to be designed as an extensible platform to train data scientists and support data-driven collaborations for interdisciplinary teams.

iPlant’s CI is a synthesis of tools and technologies from multiple, long-standing projects. While these projects provide unique foundational capabilities, they also pose unique limitations and challenges: software version dependencies, funding life cycle, and technology restrictions. However, standing on the shoulders of these giants and leveraging their best practices, collective wisdom, and experiences has allowed iPlant to rapidly build and deploy its own CI. The guiding principal for iPlant CI is best summarized by an African proverb: “If you want to go fast, go alone. If you want to go far, go together.” iPlant is committed to going farther with the help of many researchers and communities. All software developed by iPlant is available on GitHub https://github.com/iPlantCollaborativeOpenSource. Please visit iPlant at http://www.iplantcollaborative.org to get started.

## Abbreviations

API: Application Programming Interface
CI: cyberinfrastructure
CISE: Computer Information Science and Engineering
DE: Discovery Environment
DOI: digital object identifier
NGS: Next Generation Sequencing
NSF: National Science Foundation
SOPs: Standard Operating Procedures
SRA: Short Read Archive
SSO: single sign-on
VM: virtual machines
